# Evaluation of the Use of Dual Antiplatelet Therapy beyond the First Year after Acute Coronary Syndrome

**DOI:** 10.3390/jcm11061680

**Published:** 2022-03-17

**Authors:** Clara Bonanad, Sergio Raposeiras-Roubin, Sergio García-Blas, Iván Núñez-Gil, Carlos Vergara-Uzcategui, Pablo Díez-Villanueva, Jordi Bañeras, Clara Badía Molins, Jaime Aboal, Jose Carreras, Vicente Bodi, Ana Gabaldón-Pérez, Gemma Mateus-Porta, Jose Antonio Parada Barcia, Manuel Martínez-Sellés, Francisco Javier Chorro, Albert Ariza-Solé

**Affiliations:** 1 Cardiology Department, Hospital Clínico Universitario de Valencia, 46010 Valencia, Spain; clarabonanad@gmail.com (C.B.); sergiogarciablas@gmail.com (S.G.-B.); vicente.bodi@uv.es (V.B.); anagabaldonperez@gmail.com (A.G.-P.); francisco.j.chorro@uv.es (F.J.C.); 2 Department of Cardiology, INCLIVA Biomedical Research Institute, 46010 Valencia, Spain; 3 Department of Medicine, University of Valencia, 46010 Valencia, Spain; 4 Cardiology Department, Hospital Universitario Álvaro Cunqueiro de Vigo, 36213 Vigo, Spain; raposeiras26@hotmail.com (S.R.-R.); chechocat94@gmail.com (J.A.P.B.); 5 Cardiology Department, Hospital Clínico San Carlos, 28040 Madrid, Spain; ibnsky@yahoo.es (I.N.-G.); carting1@gmail.com (C.V.-U.); 6 Cardiology Department, Hospital Universitario de la Princesa, 28006 Madrid, Spain; pablo_diez_villanueva@hotmail.com; 7 Cardiology Department, Hospital Universitari Vall d’Hebrón, 08035 Barcelona, Spain; j_o_r_d_i_b@hotmail.com (J.B.); badiamolins@gmail.com (C.B.M.); 8 Cardiology Department, Hospital Josep Trueta, 17007 Girona, Spain; jaimeaboal@gmail.com; 9 Cardiology Department, Hospital del Mar, 08003 Barcelona, Spain; jcarrerasmora@psmar.cat; 10 Cardiology Department, Hospital Universitari de Bellvitge, L’Hospitalet de Llobregat, 08907 Barcelona, Spain; gemmamateus@hotmail.com; 11 Bioheart Grup de Malalties Cardiovasculars, Institut d’Investigació Biomèdica de Bellvitge—IDIBELL, L’Hospitalet de Llobregat, 08908 Barcelona, Spain; 12 Cardiology Department, Hospital Universitario Gregorio Marañón, 28007 Madrid, Spain; mmselles@secardiologia.es

**Keywords:** dual antiplatelet therapy, acute coronary syndrome, ischemic risk

## Abstract

Clinical practice guidelines recommend extending dual antiplatelet therapy (DAPT) beyond 1 year after acute coronary syndrome (ACS) in patients with high ischemic risk and without high bleeding risk. The aim of this study was to identify variables associated with DAPT prolongation in a cohort of 1967 consecutive patients discharged after ACS without thrombotic or hemorrhagic events during the following year. The sample was stratified according to whether DAPT was extended beyond 1 year, and the factors associated with this strategy were analyzed. In 32.2% of the patients, DAPT was extended beyond 1 year. Overall, 770 patients (39.1%) were considered candidates for extended treatment based on PEGASUS criteria and absence of high bleeding risk, and DAPT was extended in 34.4% of them. The presence of a PEGASUS criterion was associated with extended DAPT in the univariate analysis, but not history of bleeding or a high bleeding risk. In the multivariate analysis, a history of percutaneous coronary intervention (odds ratio (OR) = 1.8, 95% confidence interval (CI) 1.4–2.4), stent thrombosis (OR = 3.8, 95% CI 1.7–8.9), coronary artery disease complexity (OR = 1.3, 95% CI 1.1–1.5), reinfarction (OR = 4.1, 95% CI 1.6–10.4), and clopidogrel use (OR = 1.3, 95% CI 1.1–1.6) were significantly associated with extended use. DAPT was extended in 32.2% of patients who survived ACS without thrombotic or hemorrhagic events. This percentage was 34.4% when the candidates were analyzed according to clinical guidelines. Neither the PEGASUS criteria nor the bleeding risk was independently associated with this strategy.

## 1. Introduction

The prognosis of patients with acute coronary syndrome (ACS) has improved in recent decades due to advances in medical treatment, revascularization strategies, and secondary prevention measures [1]. The period of greatest risk is the first year after ACS during which the recommended medical treatment includes dual antiplatelet therapy (DAPT) [2,3]. After this first year, there is still a medium- to long-term residual risk of serious cardiovascular events (about 20%) between years 1 and 4 of follow-up after ACS [4,5].

One of the strategies aimed at improving the prognosis of patients with ACS beyond 1 year is extended DAPT. The DAPT trial showed a decrease in ischemic events in patients who received extended DAPT beyond 1 year versus discontinuation at 12 months [6]. More recently, the PEGASUS-TIMI 54 study studied patients who had an acute myocardial infarction (AMI) between 1 and 3 years before trial enrollment and presented one of the following characteristics: >65 years of age, a second infarction, diabetes mellitus, multivessel disease, or renal failure [7]. A reduction in the risk of ischemic events was observed in patients receiving long-term DAPT with ticagrelor versus aspirin in monotherapy [7]. Clinical practice guidelines recommend extended DAPT for patients with high ischemic risk and without high bleeding risk based on this evidence [2,3,8,9].

Data on the potential applicability of this recommendation in our setting are very limited. A large number of patients could benefit from prolonging DAPT following AMI based on the established criteria. Marrugat et al. used a historical cohort to estimate that more than 50% of patients ≥50 years old without bleeding episodes in the first year after AMI could benefit from this strategy [10]. However, scant information is available on its use in routine clinical practice.

The aim of this study was to evaluate for the first time in our setting the proportion of patients among the total number of potential candidates who actually receive extended DAPT, to describe their characteristics, and to identify the variables associated with extended treatment.

## 2. Materials and Methods

This was a retrospective, multicenter, observational registry study conducted in 10 hospitals in Spain. Consecutive patients discharged after ACS who received DAPT between 1 January 2017 and 31 December 2018 and who did not present thrombotic or hemorrhagic events during the first year were included. Antiplatelet therapy parameters during follow-up were evaluated. All data were collected and processed by assigning each patient a code for anonymous data management in accordance with applicable state legislation. The entire process also complied with ethical and legal standards, in particular the Declaration of Helsinki and the Oviedo Convention, and good clinical practice guidelines for research on human beings. The study was approved by the Ethics Committee of the reference hospital, as well as those of the participating hospitals.

### 2.1. Study Population

The study population comprised patients who were candidates for extended DAPT beyond 1 year after ACS that was revascularized using percutaneous coronary intervention (PCI). We included patients who were discharged after ACS and who met the following criteria: obstructive coronary artery disease (defined as stenosis ≥50% of the left coronary artery and ≥70% of any other artery, or invasive evidence of ischemia) diagnosed using coronary angiography at admission, PCI performed, treatment at discharge with DAPT, and survival > 1 year after the initial event. Patients with an indication for oral anticoagulation at discharge or if this indication emerged during the first year or who developed any thrombotic, hemorrhagic, or transfusion-related events during the first year after discharge were excluded.

The sample was stratified according to whether DAPT was extended beyond 1 year after ACS. The logistics of outpatient follow-up often do not allow for the decision on the maintenance or suspension of DAPT for all cases to be taken exactly at the end of 12 months; therefore, we decided to accept a margin of 2 months. Thus, if DAPT was discontinued before 14 months (425 days), it was considered to be discontinued within 1 year, and extended if it was discontinued at any time after that point.

### 2.2. Study Variables and Follow-Up

Demographic, clinical, analytical, and echocardiographic data; characteristics of coronary disease; and treatment at discharge were collected for each patient. Regarding coronary artery disease, data regarding the left main, proximal left anterior descending artery (LAD), or multivessel disease (i.e., at least two coronary territories affected) were collected. The presence of at least 1 PEGASUS clinical trial criterion (>65 years old, a second infarction, diabetes mellitus, multivessel disease, or renal failure) in each patient was determined and the total number of criteria was recorded. Any history of bleeding, recurrent bleeding, and intracranial bleeding was collected. Patients were considered to have a high bleeding risk if they had at least 1 major criterion or 2 minor criteria for high bleeding risk according to the Academic Research Consortium (ARC-HBR) [11]. The time of DAPT discontinuation was recorded during the maximum follow-up period available for each patient. The collection of events and changes in treatment during follow-up was carried out through an electronic medical record managed by an expert data manager.

### 2.3. Objectives

The aim of the study was to evaluate the proportion of patients who continued DAPT beyond 1 year after ACS and the predictors of prolonged therapy. Specifically, our aim was to determine the impact of the PEGASUS criteria on the decision to prolong DAPT in our setting.

### 2.4. Statistical Analysis

In the descriptive analysis, qualitative variables were presented as absolute values and percentages. For quantitative variables, the presence of a normal distribution was assessed using the Kolmogorov–Smirnov test, and the mean and standard deviation were expressed if the distribution was normal; otherwise, the median and interquartile range were presented.

The association of each of the study variables with extended APT was evaluated using the χ^2^ method (chi-square) for qualitative variables and Student’s *t*-test for quantitative variables, defining a value of *p* < 0.05 as significant. All variables showing significant association in a univariate analysis were then evaluated in a multivariate analysis using logistic regression. The results are expressed using odds ratios, 95% confidence intervals, and *p*-values. Variables related (*p* < 0.05) with extended APT based on a univariate analysis were used in multivariate analysis with logistic regression. A backward stepwise procedure was subsequently used to identify independent related variables constructing a parsimonious multivariate model. The usefulness of which was evaluated using the area obtained under the ROC curve and the overall performance of the model (correctly classified cases/total cases).

## 3. Results

In total, 1967 patients were included, with a mean age of 64.5 years (standard deviation 12.8 years); 77.3% were men. The index event in almost half (49.1%) of the patients was ST-segment elevation ACS. The most frequently indicated P2Y12 blocker was ticagrelor, used in 58.8% of patients, followed by clopidogrel in 30.3%, and prasugrel in 11.0%. Baseline patient characteristics are shown in Table 1.

### 3.1. DAPT Duration

**Figure 1:**
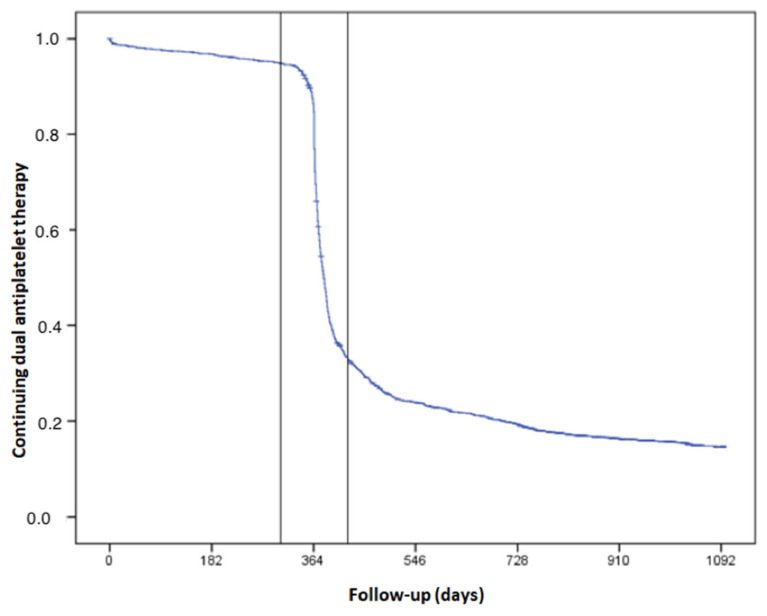
Patients that continued DAPT beyond 1 year after ACS, taking into account a margin of ±2 months between month 10 (day 205) and month 14 (day 425).

**Figure 2:**
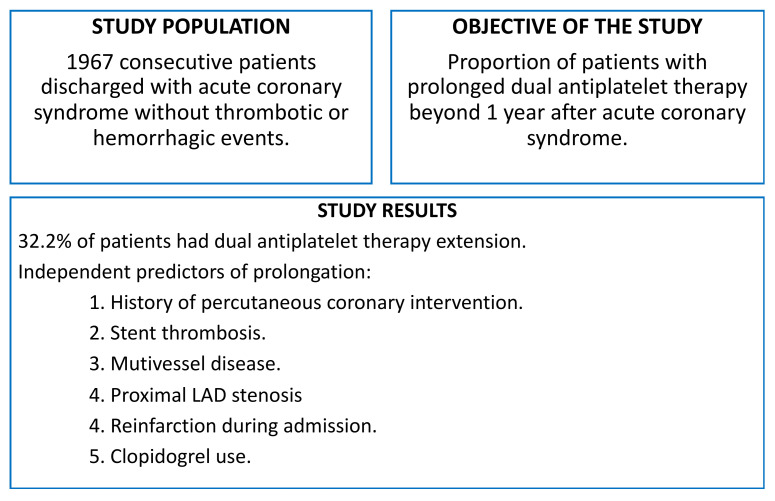
Overall summary of included patients to whom dual antiplatelet therapy was extended. LAD: left anterior descending.

Overall, 94.0% of patients continued to receive DAPT at 10 months (day 305), while 32.2% continued to receive DAPT at 14 months (day 425) (Figure 1 and Figure 2). The latter cases were defined as extended DAPT. At 2 years after the index event, 19.3% continued DAPT therapy.

### 3.2. Criteria for Candidates for Extended DAPT

When evaluating the criteria to be met to establish a candidate for extended DAPT, 71.8% of patients were found to meet at least one PEGASUS criterion. On the other hand, 36.5% of the total series had a high bleeding risk according to the ARC-HBR criteria, while 45% of patients with at least one PEGASUS criterion had a high bleeding risk. Ultimately, a total of 770 patients (39.1%) were estimated to be candidates for extending DAPT beyond 1 year based on the presence of a PEGASUS criterion and absence of high bleeding risk. DAPT was extended in 34.4% of these potential candidates.

#### 3.2.1. Univariate Analysis

Table 2 shows the results of the univariate analysis of extended DAPT. When the clinical history was analyzed, a significant association was found for hypertension, diabetes mellitus, dyslipidemia, peripheral arterial disease, heart failure, previous PCI, stent thrombosis, stroke, and dialysis. With regard to the index ACS, the ACS type, Killip class, reinfarction, and creatinine levels were associated. For the characteristics of coronary disease, involvement of the coronary left main, proximal anterior descending coronary artery, and multivessel disease were significantly associated, while the number, length, and diameter of implanted stents had no influence. Furthermore, an association was shown between ACS treatment and beta blockers, angiotensin-converting-enzyme inhibitors, angiotensin II receptor antagonists, and the type of second antiplatelet agent used.

Finally, statistically significant differences were observed in the prolongation of DAPT at the different hospitals, ranging from 14.2% to 52.5%.

The presence of at least one criterion from the PEGASUS clinical trial was significantly associated in the univariate analysis with extending DAPT, but the total number of such criteria was not. Neither the history of bleeding (whether overall or recurrent or intracranial) nor a high bleeding risk according to the ARC-HBR criteria was associated with extending DAPT. A high bleeding risk also had no influence on extending DAPT in patients with PEGASUS criteria.

#### 3.2.2. Multivariate Analysis

In the multivariate analysis with logistic regression, the variables that showed statistically significant associations with extending DAPT were a history of PCI, stent thrombosis, proximal LAD stenosis, multivessel disease, reinfarction during index admission, and clopidogrel use (versus ticagrelor or prasugrel) (Table 3). The overall performance of the model was 69.6%, with an area under the ROC curve of 0.623.

Finally, an additional multivariate analysis was performed to identify variables associated with extended DAPT in the subgroup of patients treated with ticagrelor. Previous PCI, previous coronary artery disease, type of acute coronary syndrome, proximal LAD stenosis, multivessel disease, and reinfarction during index admission were independently associated with prolongation of DAPT. Although univariate analysis found that the presence of at least one PEGASUS criterion was significantly associated with extending DAPT, this was not confirmed in the multivariate model.

## 4. Discussion

The results of this registry study showed that DAPT was extended beyond 1 year in 32.2% of patients who survived ACS without ischemic or bleeding events. This percentage was similar (34.4%) when the subgroup of patients who were candidates for prolonging DAPT was analyzed according to the clinical guidelines, defined as cases with ischemic risk criteria (PEGASUS trial criteria in this case) and no high bleeding risk.

In this population, up to 76% of patients had some PEGASUS clinical trial criteria; however, neither the presence of a PEGASUS criterion nor the presence of a high bleeding risk showed a significant influence on the prolongation of DAPT. Independent predictive factors associated with DAPT prolongation were a history of PCI, stent thrombosis, proximal LAD stenosis, multivessel disease, reinfarction during index admission, and clopidogrel use.

Two clinical trials provided the main evidence for extending DAPT beyond 1 year after ACS. The DAPT trial, published in 2014, showed a decrease in the composite of death, infarction or stroke, and stent thrombosis in patients who extended DAPT beyond 1 year (up to 30 months) after stent revascularization compared to those who discontinued at 12 months [6]. In contrast, increased bleeding and increased non-cardiovascular mortality were observed with long-term DAPT. When indications were analyzed, the subgroup of patients with AMI as the reason for revascularization showed the greatest benefit in terms of ischemic events [6]. The PEGASUS-TIMI 54 study, published in 2015, included patients who had an AMI between 1 and 3 years before inclusion and had at least 1 other risk factor (>65 years old, a second infarction, diabetes mellitus, multivessel disease, or renal failure). Participants were randomized to aspirin monotherapy or DAPT consisting of aspirin plus ticagrelor at doses of 90 mg/12 h or 60 mg/12 h. The primary study endpoint was reached with the reduction of the risk of cardiovascular death, infarction, or stroke among patients who received ticagrelor, although there was an increase in major bleeding, but not intracranial hemorrhage or fatal bleeding [7]. The most favorable net benefit was observed with the 60 mg dose of ticagrelor.

Since the publication of both studies, the recommendation to extend DAPT beyond 1 year after ACS was included in various clinical practice guidelines of the European Society of Cardiology: ACS with ST-segment elevation in 2017 (grade of recommendation IIb, level of evidence B), DAPT in 2017 (IIb, A), myocardial revascularization in 2018 (IIb, A), and ACS without ST-segment elevation in 2020 (IIa, A) [2,3,8,9]. Despite this, scant data are available on the implementation of this strategy in routine clinical practice. The PARIS registry (Patterns of Non-Adherence to Antiplatelet Regiments in Stented Patients) found a rate of DAPT of 37.2% at 2 years after ACS treated by PCI in a cohort of 2056 patients recruited between 2009 and 2010 [12]. The EPICOR (Long-Term Follow-Up of Antithrombotic Management Patterns in Acute Coronary Syndrome Patients; NCT01171404) registry showed DAPT continuation at 2 years in 57% of patients who had ACS between 2010 and 2011, although this excluded patients who died or were lost to follow-up. Statistically significant differences in rates between countries are notable [13]. The analysis of the 782 patients included by hospitals in Spain in the EPICOR registry showed a rate of prolongation of DAPT of 41.4% [14]. Data from these registries come from cohorts recruited before the publication of clinical trials and the implementation of new clinical practice guideline recommendations. More recently, the Italian STAR ANTIPLATELET registry included 596 patients who in 2017 had completed 12 months of DAPT following ACS; it was found that this treatment had been extended for 13% of patients [15]. It should be noted that this study was conducted before the use of ticagrelor beyond 1 year was approved in Italy. In Spain, Marrugat et al. used data from a historical cohort of the REGICOR registry to estimate that the number of patients who met at least one PEGASUS criterion was more than 22,000 patients per year, i.e., over 50% of patients ≥50 years old without bleeding events in the first year after AMI [10].

Unlike the registries cited, our study included a contemporary cohort (2017–2018), and we believe that our figures reflected the impact of clinical trials, clinical practice guideline recommendations, and the marketing of the 60 mg dose of ticagrelor in routine clinical practice. To obtain a more accurate picture of the prolongation of DAPT, we also decided to exclude patients who had an ischemic or bleeding event during the first year from the analysis since this fact inevitably altered the approach to long-term DAPT. Taking these premises into account, we found that 32.2% of patients received DAPT that was extended beyond 1 year. To try to estimate whether that percentage is accurate in the light of current evidence and recommendations, we defined the ideal candidate for extending DAPT as one with at least one PEGASUS test criterion who did not present a high bleeding risk as defined by the HBR criteria. Overall, 770 patients (39.1%) met this definition of which only 34.4% received extended therapy, demonstrating that this strategy is clearly underused.

The results of our study show an association between the presence of a PEGASUS clinical trial criterion and the prolongation of DAPT. However, this finding is not independently confirmed in the multivariate analysis. Similar findings were obtained analyzing only the subgroup of patients receiving ticagrelor. Surprisingly, the presence of a high bleeding risk had no influence on the duration of DAPT in our cohort. Therefore, the decision to implement this strategy did not appear to correspond to the criteria set out in the clinical guidelines. Similarly, in the STAR ANTIPLATELET registry, neither the thrombotic risk (estimated from the atherothrombotic risk score derived from the TRA 2 × P study) nor the bleeding risk (PRECISE-DAPT) was associated with extended DAPT, although the absence of anemia and bleeding was. In the analysis of the Spanish cohort of the EPICOR registry, the proportion of patients who continued DAPT at 2 years was similar in patients who had an ischemic event and in those who had a bleeding event [14,15]. However, our data contrasted with the observation that the bleeding risk influenced the choice of antiplatelet therapy following ACS: indeed, lower scores on the CRUSADE scale were associated with the use of more potent P2Y12 blockers (ticagrelor and prasugrel) [16].

It is therefore of particular interest to analyze which factors lead to the choice of prolonging DAPT in these patients. Our study showed that a history of PCI or stent thrombosis, along with proximal LAD stenosis, multivessel disease, reinfarction during index admission, and clopidogrel use, would be factors that lead to choosing to extend DAPT. This is probably because the perception of a higher ischemic risk due to the previous recurrence of clinical events and the coronary disease characteristics are underlying factors in deciding whether to prolong DAPT in routine clinical practice in our setting. The STAR ANTIPLATELET registry also found that recurrent ischemic events were one of the independent predictors of prolongation of therapy, along with renal failure, peripheral artery disease, and the absence of anemia or hemorrhage [15]. The complexity of coronary artery disease is recognized as a high-risk criterion of ischemic events and it may support DAPT extension [3]. Recent ESC guidelines for the management of acute coronary syndromes in patients presenting without persistent ST-segment elevation include a series of risk criteria for extended treatment with a second antithrombotic agent based on the combined evidence of clinical trials and registries addressing this topic [3]. Stent thrombosis, recurrent myocardial infarction, and multivessel coronary disease are the main variables independently associated with extended DAPT in our study, and all of them are included in the aforementioned risk criteria. These variables easily identify a high-risk profile; therefore, it is not surprising that they are the most used in clinical daily practice in our study. However, this approach excludes other variables included both in the PEGASUS criteria and in the ESC guidelines that also pose a higher risk. Therefore, the decision to extend DAPT in clinical practice seems to rely on some important variables but ignore other relevant factors and the result is the underuse of this strategy.

It is paradoxical to find that patients treated with clopidogrel more often receive extended DAPT than patients receiving ticagrelor or prasugrel. Different factors could contribute to this finding, such as the perception of increased bleeding risk with prasugrel or ticagrelor or more demanding administrative requirements for the prescription of these drugs. Both situations would generate therapeutic inertia unrelated to the clinical criteria. In any case, this observation reinforces the impression that the PEGASUS strategy is poorly implemented in clinical practice in our setting.

Finally, it should be noted that the overall performance of the multivariate model was modest, which indicated that there may be variables that were not considered in our study that influence the prolongation or non-prolongation of DAPT in clinical practice or that perhaps some arbitrary factors are at play. Along this line, it is interesting to analyze the differences between the different recruitment centers. Significant variations were observed in the prolongation of DAPT at the different hospitals, with rates ranging from 14.2% to 52.5%. Since our study was based on a registry of consecutive patients, these differences were unlikely to be due to the characteristics of the patients included, but are probably more closely related to local protocols or peculiarities, such as ease of prescription and prescriber preferences. The multivariate analysis did not confirm an independent association with the hospital where the patient was recruited, but it did not rule out that certain, sometimes intangible, reasons may explain more or less use of extended DAPT. In a similar vein, as noted above, the EPICOR register showed statistically significant differences in DAPT rates between countries [13].

Our study had some limitations. First, this was a retrospective observational registry study; therefore, the presence of confounding factors that remained unanalyzed is likely, possibly due to the existence of specific local protocols. Some of the variables, such as hemoglobin or creatinine levels, were collected at admission, but changes during follow-up (not included in the analysis) may have influenced the decision to prolong DAPT. The use of a validated score to assess coronary complexity may have provided additional useful information. Patients with an indication for oral anticoagulation and those who had ischemic or bleeding events during the first year were excluded; therefore, our series did not reflect the entire population. Nevertheless, this selection helped to define the proportion of patients who are candidates for extended DAPT, the implementation of this strategy in daily clinical practice, and predictors of prolongation with much greater precision. Finally, the study lacked statistical power to compare clinical events between treatment strategies (i.e., 12 months vs. extended DAPT).

Despite these limitations, in our view, this study is the first reliable description of the application of the recommendation to prolong DAPT beyond 1 year in patients with ischemic risk but without high bleeding risk in daily clinical practice in our setting. Proper adherence to recommendations could lead to a significant reduction in ischemic events and the associated clinical, economic, and social consequences in these patients.

## 5. Conclusions

About three out of every four patients with ACS who were not candidates for oral anticoagulation and who did not present ischemic or bleeding events beyond 1 year of admission had some PEGASUS criteria for ischemic risk. Neither the presence of PEGASUS criteria nor the bleeding risk was independently associated with extended DAPT. Independent predictors of extending DAPT were a history of PCI, stent thrombosis, proximal LAD stenosis, multivessel disease, reinfarction during index admission, and clopidogrel use.

## Figures and Tables

**Table 1:** Patients’ basal characteristics.

Demographics		
Female sex, % (*n*)		21.4% (421)
Age, years		64.5 ± 12.8
**Cardiovascular risk factors**		
Smoking	No, % (*n*)	36.6% (720)
	Former smoker	25.3% (498)
	Current smoker	38.1% (749)
Hypertension, % (*n*)		57.7% (1135)
Dyslipidemia, % (*n*)		54.7% (1076)
Diabetes mellitus	No, % (*n*)	72.6% (1428)
	Type I, % (*n*)	0.6% (11)
	Type II, % (*n*)	26.8% (528)
**Cardiac antecedents and comorbidities**		
Heart failure, % (*n*)		2.7% (53)
Coronary artery disease, % (*n*)		48.0% (945)
PCI, % (*n*)		14.2% (279)
Stent thrombosis, % (*n*)		1.8% (36)
Peripheral arterial disease, % (*n*)		7.8% (153)
Stroke, % (*n*)		4.7% (93)
Bleeding, % (*n*)		1.6% (32)
Active cancer, % (*n*)		3.3% (65)
Dialysis, % (*n*)		1.2% (24)
Cirrhosis, % (*n*)		0.4% (8)
**PEGASUS Criteria and High Bleeding Risk**		
Meets PEGASUS criteria, % (*n*)		71.8% (1400)
High bleeding risk, % (*n*)		36.3% (715)
PEGASUS criterion and no high bleeding risk, % (*n*)		39.5% (770)
**Acute coronary syndrome**		
ACS type	Unstable angina, % (*n*)	12.5% (245)
	NSTEMI, % (*n*)	38.0% (747)
	STEMI, % (*n*)	49.1% (965)
Killip > I, % (*n*)		11.6% (228)
Coronary left main disease, % (*n*)		7.0% (138)
Proximal LAD disease, % (*n*)		33.6% (660)
Multivessel disease, % (*n*)		52.3% (1029)
Incomplete revascularization, % (*n*)		21.6% (424)
**Additional tests during admission**		
Creatinine, mg/dL		0.80 ± 0.68
Platelets, cells/μL		227,147 ± 70,086
Leukocytes, cells/μL		10,401 ± 3614
LVEF, %		54.7 ± 9.6
**Complications during admission**		
Reinfarction, % (*n*)		1.1% (22)
Major bleeding, % (*n*)		1.1% (22)
Heart failure, % (*n*)		4.0% (79)
**Treatment at discharge**		
ASA, % (*n*)		99.2% (1951)
P2Y12 inhibitor	Clopidogrel, % (*n*)	30.3% (592)
	Ticagrelor, % (*n*)	58.8% (1150)
	Prasugrel, % (*n*)	11.0% (215)
Betablockers, % (*n*)		84.0% (1667)
ACEI or AIIRA or ARNI, % (*n*)		74.3% (1461)
Spironolactone or eplerenone, % (*n*)		6.9% (136)
Statins, % (*n*)		97.2% (1912)

ACEI: angiotensin-converting enzyme inhibitor; ACS: acute coronary syndrome; LAD: left anterior descending artery; AIIRA: angiotensin II receptor antagonist; ARNI: angiotensin receptor neprilysin inhibitor; ASA: acetylsalicylic Acid; DAPT: dual antiplatelet therapy; LVEF: left ventricular ejection fraction; NS: non-significant; NSTEMI: non-ST segment elevation myocardial infarction ST; PCI: percutaneous coronary intervention; STEMI: ST-segment elevation myocardial infarction. Data are expressed as % (*n*) for qualitative variables and mean ± standard deviation for quantitative variables.

**Table 2:** Univariate analysis of extended DAPT.

		Non-Extended DAPT ^1^ 67.8% (*n* = 1333)	Extended DAPT ^1^	*p*-Value
**Demographics**				
Female sex, % (*n*)		21.5% (284)	22.1% (1037)	NS
Age, years		64.4 ± 12.8	64.8 ± 12.9	NS
**Cardiovascular risk factors**				
Smoking	No, % (*n*)	36.4% (484)	35.8% (225)	
	Former smoker, % (*n*)	23.9% (318)	28.7% (180)	NS
	Current smoker, % (*n*)	39.6% (526)	35.5% (223)	
Hypertension, % (*n*)			62.8% (396)	0.003
Dyslipidemia, % (*n*)			62.1% (392)	<0.001
Diabetes mellitus	No, % (*n*)	75.2% (998)	66.9% (422)	
	Type I, % (*n*)	0.3% (4)	1.1% (7)	<0.001
	Type II, % (*n*)	24.5% (326)	32.0% (202)	
**Cardiac antecedents and comorbidities**				
Heart failure, % (*n*)		1.8% (24)	4.6% (29)	<0.001
Coronary artery disease, % (*n*)		50.4% (945)	43.9% (277)	0.007
PCI, % (*n*)		10.4% (139)	22.1% (140)	<0.001
Stent thrombosis, % (*n*)		0.7% (9)	4.3% (27)	<0.001
Peripheral arterial disease, % (*n*)		6.4% (85)	10.7% (68)	0.001
Stroke, % (*n*)		4.1% (54)	6.2% (39)	0.041
Bleeding, % (*n*)		0.9% (12)	0.8% (5)	NS
Active cancer, % (*n*)		3.5% (47)	2.9% (18)	NS
Dialysis, % (*n*)		1.2% (16)	1.3% (8)	NS
Cirrhosis, % (*n*)		0.4% (5)	0.5% (3)	NS
**PEGASUS Criteria and High Bleeding Risk**				
Meets PEGASUS criteria, % (*n*)		69.9% (924)	75.8% (476)	0.007
High bleeding risk, % (*n*)		36.2% (481)	36.6% (232)	NS
PEGASUS criterion and no high bleeding risk, % (*n*)		38.2% (505)	42.2% (265)	NS
**Acute coronary syndrome**				
ACS type	Unstable angina, % (*n*)	13.3% (176)	10.9% (69)	0.010
	NSTEMI, % (*n*)	35.9% (476)	42.9% (271)	
	STEMI, % (*n*)	50.8% (673)	46.2% (292)	
Killip > I, % (*n*)		10.2% (134)	14.9% (94)	0.002
Coronary left main disease, % (*n*)		6.1% (81)	9.0% (57)	0.018
Proximal LAD disease, % (*n*)		30.1% (401)	41.1% (259)	<0.001
Multivessel disease, % (*n*)		49.3% (657)	58.8% (372)	<0.001
Incomplete revascularization, % (*n*)		21.4% (286)	22.8% (142)	NS
**Additional tests during admission**				
Creatinine, mg/dL		0.73 ± 0.70	0.89 ± 0.73	<0.001
Platelets, cells/μL		225,181 ± 68,501	231,265 ± 72,179	NS
Leukocytes, cells/μL		10,433 ± 3637	10,333 ± 3567	NS
LVEF, %		54.6 ± 9.5	54.9 ± 9.8	NS
**Complications during admission**				
Reinfarction, % (*n*)		0.5% (7)	2.4% (15)	<0.001
Major bleeding, % (*n*)		0.9% (12)	1.6% (10)	NS
Heart failure, % (*n*)		3.5% (46)	5.2% (33)	NS
**Discharge treatment**				
ASA, % (*n*)		99.3% (1320)	98.9% (621)	NS
P2Y12 inhibitor	Clopidogrel, % (*n*)	27.9% (371)	35.2% (221)	0.004
	Ticagrelor, % (*n*)	60.5% (804)	55.1% (346)	
	Prasugrel, % (*n*)	11.6% (154)	9.7% (61)	
Betablockers, % (*n*)		83.3% (1105)	87.5% (548)	0.015
ACEI or AIIRA or ARNI, % (*n*)		72.3% (957)	77.8% (487)	0.009
Spironolactone or eplerenone, % (*n*)		6.3% (83)	8.5% (53)	NS
Statins, % (*n*)		97.1% (1289)	97.3% (609)	NS

^1^ Considered prolonged if not discontinued at 1 year (± 2 months). ACEI: angiotensin-converting enzyme inhibitor; ACS: acute coronary syndrome; LAD: left anterior descending artery; AIIRA: angiotensin II receptor antagonist; ARNI: angiotensin receptor neprilysin inhibitor; ASA: acetylsalicylic acid; DAPT: dual antiplatelet therapy; LVEF: left ventricular ejection fraction; NS: non-significant; NSTEMI: non-ST segment elevation myocardial infarction ST; PCI: percutaneous coronary intervention; STEMI: ST-segment elevation myocardial infarction. Data are expressed as % (*n*) for qualitative variables and mean ± standard deviation for quantitative variables.

**Table 3:** Multivariate analysis that shows the variables that were associated with extending DAPT.

	OR	OR 95% CI	*p*-Value
History of PCI	1.847	1.395–2.445	<0.001
History of stent thrombosis	3.854	1.661–8.943	0.002
Proximal LAD stenosis	1.444	1.177–1.771	<0.001
Multivessel disease	1.266	1.036–1.546	0.021
Reinfarction during admission	4.117	1.636–10.363	0.003
Clopidogrel at discharge ^1^	1.332	1.081–1.641	0.007

Odds ratio and its confidence interval are for each point. ^1^ Versus ticagrelor or prasugrel. CI: confidence interval; DAPT: dual antiplatelet therapy; OR: odds ratio; PCI: percutaneous coronary intervention; LAD: left anterior descending artery.

## Data Availability

Data are available upon requested to the corresponding author.
